# An unusual timing for symptomatic chest pain in an adult chest wall myofibroma: a case report

**DOI:** 10.1186/1752-1947-8-210

**Published:** 2014-06-19

**Authors:** Chin-Li Chen, Hung Chang

**Affiliations:** 1 Division of Thoracic Surgery, Department of Surgery, Tri-Service General Hospital, National Defense Medical Center, No.325, Section 2, Cheng-Kung Road, Taipei 114, Taiwan

**Keywords:** Chest pain, Chest wall tumor, Myofibroma, Thoracotomy

## Abstract

**Introduction:**

Myofibromas are benign mesenchymal neoplasms that can present as solitary and multicentric lesions. They can occur in several locations and can occur at any age from neonates to elderly patients. However, most of the lesions are found in neonates and babies. It rarely occurs in adults.

**Case presentation:**

A 29-year-old Taiwanese man presented with persistent dull chest pain in his right lateral chest wall for 2 weeks. A chest X-ray showed a faint patchy opacity over the periphery of his right upper lung zone. Computed tomography and magnetic resonance imaging showed a lobulated mass at the intercostal space between his right fifth and sixth ribs with contrast enhancement and bone invasion. Malignancy could not be excluded. A percutaneous needle aspiration biopsy failed due to technique issues, so he underwent a thoracotomy and the tumor was excised with Marlex mesh repairs for the thoracic defect. Pathology confirmed a myofibroma without malignancy. He recovered uneventfully and no local recurrence was detected at the 1-year follow-up examination.

**Conclusions:**

Chest wall myofibroma presenting with chest pain has never been reported in adults. It is a challenge to differentiate myofibroma from malignancy in chest wall preoperatively, such as seen in our patient. To the best of our knowledge, this has not been previously reported in the scientific literature. Although myofibroma rarely occurs in the chest wall and adults, it must be suspected in any chest wall tumor presenting with chest pain.

## Introduction

Myofibroma is a benign mesenchymal neoplasm composed of myofibroblasts. Most myofibromas consist of a firm, well-circumscribed mass and grow slowly. Many terms have been used for this neoplasm since the first report in 1951, including “benign neoplasm” and “malignant neoplasm” [1]. Consequently, it is easily misdiagnosed. Myofibromas can occur in several locations including skin, subcutaneous tissues, skeletal muscle, oral cavity, head and extremities [2]. Clinically, myofibromas most commonly present in children younger than 2-years old and two-thirds of these lesions present at birth [2]. Myofibromas rarely occur in adults and a chest wall myofibroma has never been reported in an adult. To the best of our knowledge, this is the first documented case of a chest wall myofibroma in an adult and initially it is a challenge to differentiate from a malignancy.

## Case presentation

**Figure 1:**
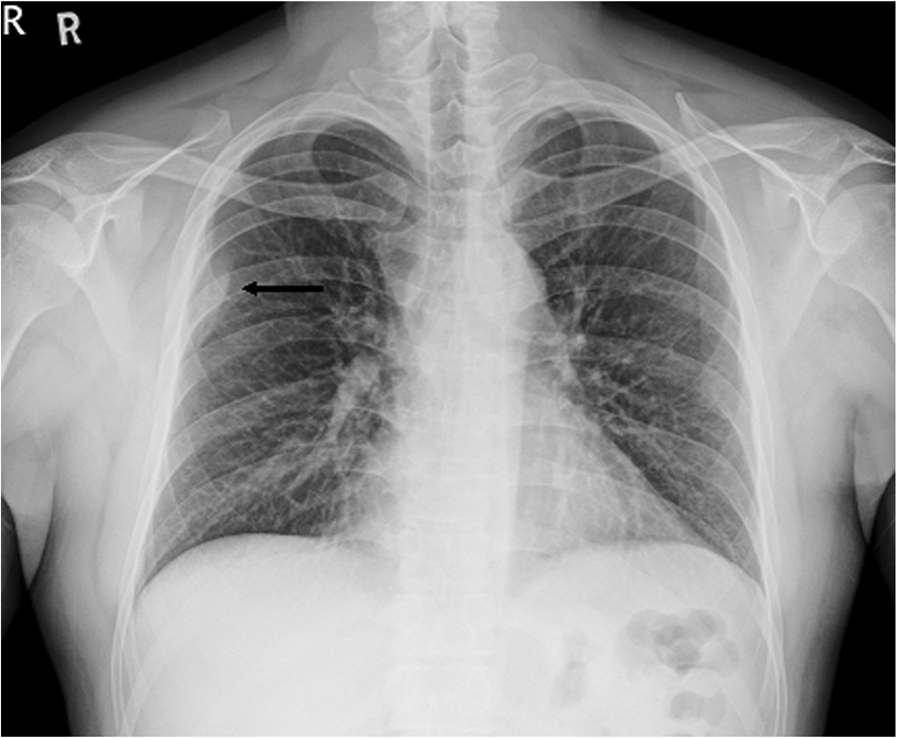
**Chest X-ray of the patient.** A chest X-ray showed a faint patchy opacity over the periphery of the right upper lung zone (black arrow).

**Figure 2:**
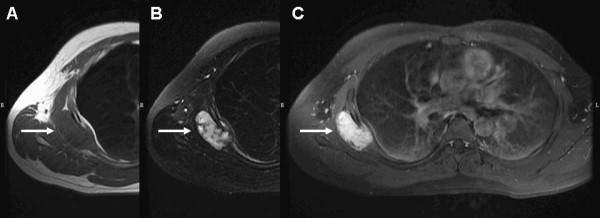
**Magnetic resonance imaging of chest shows a 5.2×4.5×4.6cm mass involving the right serratus anterior muscle, the right fifth intercostal space and the subpleural space (white arrow). (A)** T1-weighted image shows isointense to hypointense relative to the muscle. **(B)** T2-weighted image shows heterogeneously hyperintense with some low-signal spots. **(C)** T2-weighted image with gadolinium injection shows inhomogeneous contrast enhancement.

**Figure 3:**
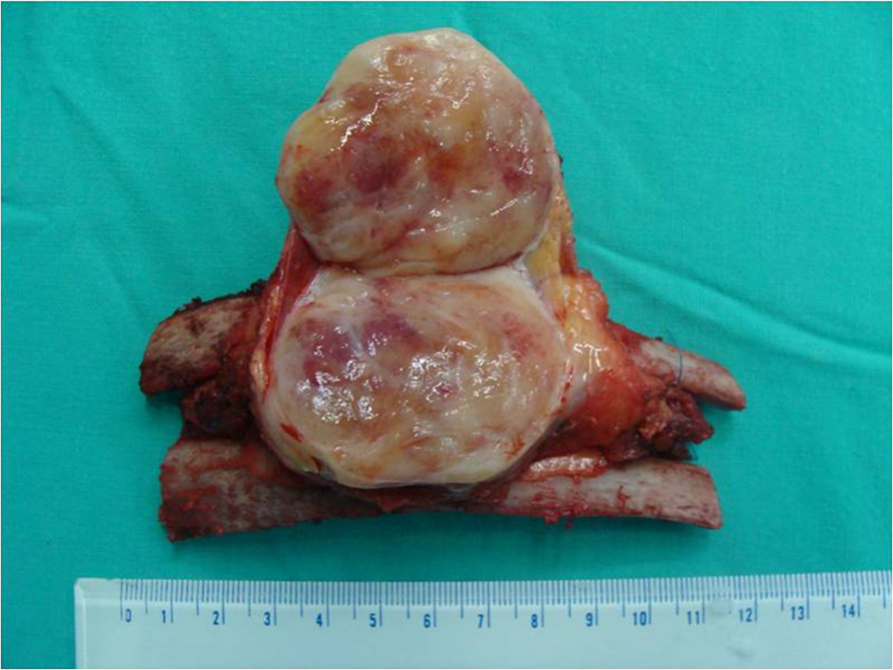
**The gross picture of the tumor.** A soft tissue mass measured approximately 5.0×5.0×4.2cm in size with a white-yellowish appearance.

**Figure 4:**
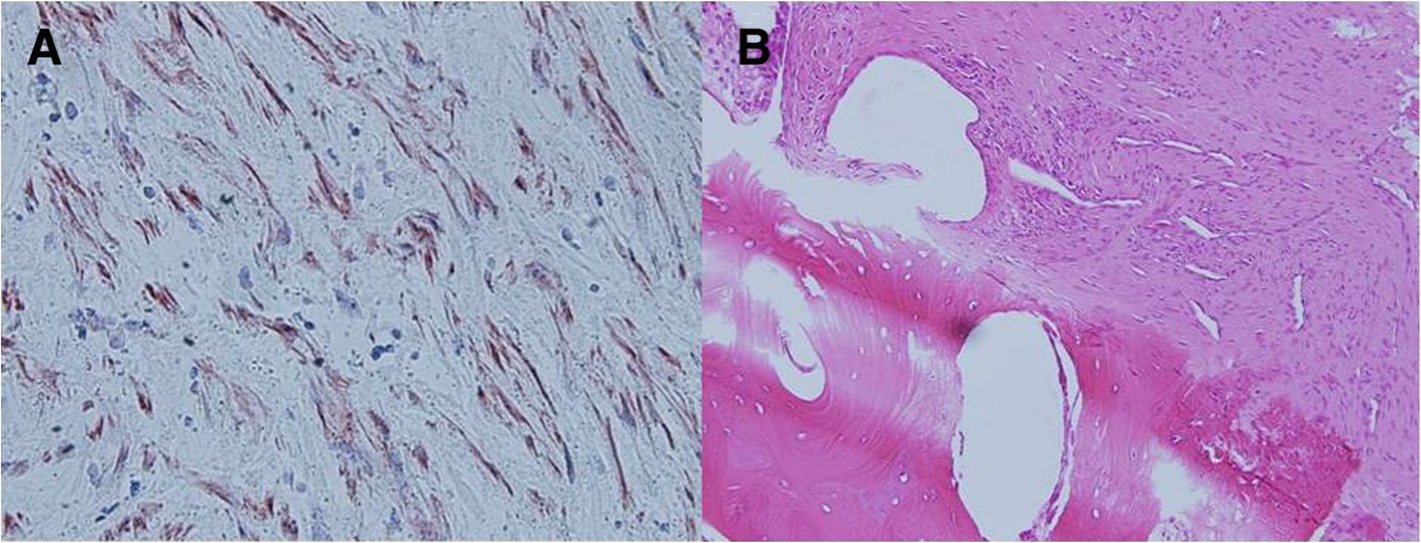
**The histopathology of the tumor. (A)** Calponin ×400: hypocellular myxoid appearance with short fascicles, tumor cells and abundant myxoid stroma. **(B)** Hematoxylin and eosin stain ×200: focal bone invasion.

A 29-year-old Taiwanese man presented to our emergency department with persistent dull pain over his right chest wall for 2 weeks. His chest pain was characterized by a persistent dull ache in his right lateral chest wall. No other constitutional symptoms were noticed. Furthermore, he did not have a palpable mass over his right chest wall and the external appearance of his body was normal. A physical examination was unremarkable except for mild tenderness over his right chest wall.A chest X-ray showed a faint patchy opacity over the periphery of his right upper lung zone (Figure 1). Chest computed tomography (CT) showed a lobulated mass approximately 5.5×4.5×4.3cm in size at the intercostal space between his right fifth and sixth ribs. Magnetic resonance imaging (MRI) of his thorax revealed a 5.2×4.5×4.6cm lobulated mass with bone invasion of the adjacent ribs (Figure 2). The mass was isointense to hypointense relative to the muscle on T1 pregadolinium, and heterogeneously hyperintense on fat-suppressed T2 pregadolinium with some low-signal spots and tube-like structures. It showed inhomogeneous contrast enhancement with gadolinium injection. Malignancy could not be excluded according to the image studies.We expected to perform a CT-guided needle aspiration biopsy initially, but it failed due to the barriers of right serratus anterior muscle, latissimus dorsi muscles and scapula. Subsequently, we performed a standard thoracotomy and excised the tumor (Figure 3). During surgery, the tumor was found not to adhere to the lung parenchyma. After separating the latissimus dorsi muscles and serratus anterior muscles, we noted a solid and firmly fixed tumor measuring 5.0×5.0×4.2cm in size over his fifth to sixth intercostal space and it could not be dissected off his fifth and sixth ribs. Subsequently, tumor excision along with segmental resection of his fifth and sixth ribs was done. The defect of his chest wall was reconstructed with Marlex mesh. Grossly the tumor was white-yellow in color and had a smooth appearance. On histological examination, the lesion presented as a hypocellular myxoid tumor composed of short fascicles, tumor cells and abundant myxoid stroma (Figure 4). Neither cytologic atypia nor pleomorphism was observed. The findings were compatible with the diagnosis of a chest wall myofibroma. After surgery, he recovered uneventfully and was discharged on postoperative day 6. No recurrence was detected at the 1-year follow-up examination.

## Discussion

Myofibroma (unifocal) and myofibromatosis (multifocal) are rare spindle-cell neoplasms composed of myofibroblasts. Typically, these lesions present in children and babies, which prompted the use of terms such as congenital fibrosarcoma, congenital generalized fibromatosis, congenital mesenchymal hamartoma and infantile myofibromatosis [2]. Neonates and babies have the highest incidence of myofibroma and myofibromatosis: 90% of myofibromatosis cases occur before the age of 2 years, and two-thirds are present at birth. Nonetheless, both solitary and multicentric lesions can occur at any age [2]. Adult symptomatic chest wall myofibroma were rarely reported.

These lesions mostly involve the head and neck, although some cases of involvement of the trunk and extremities have been reported [2]. The most common locations include skin, subcutaneous tissues, skeletal muscle and oral cavity [2]. Most myofibromas consist of a firm, well-circumscribed mass and grow slowly. The differential diagnosis includes the possibility of infections (tuberculosis or fungal growths), benign and malignant connective tissue lesions (fibroma, peripheral giant cell granuloma, fibrous histiocytoma, fibromatosis, fibrosarcoma or malignant fibrous histiocytoma), benign and malignant neural neoplasms (palisaded encapsulated neuroma, schwannoma, neurofibroma or neurofibromatosis and neurofibrosarcoma), benign and malignant muscle neoplasms (leiomyoma, rhabdomyoma, leiomyosarcoma, rhabdomyosarcoma or alveolar soft-part sarcoma), vascular lesions (hemangiomas), salivary gland pathology (for example, pleomorphic adenoma or mucoepidermoid carcinoma) and lymphoma or chloroma [2,3]. Common chest wall tumors and their related symptoms are listed in Table 1. The prognosis for most lesions is excellent, with the exception of generalized myofibromatosis, a condition associated with multicentric visceral lesions of the vital organs of babies that has an aggressive and sometimes fatal outcome [2].

**Table 1:** Chest wall tumors and related symptoms

	**Common presenting age (years old)**	**Typical symptoms**
**Myofibroma (the reported case)**	**N/A**	**N/A**
**Lipoma**	**50–70**	**An asymptomatic, well-circumscribed mobile mass**
**Multiple myeloma**	**>60**	**Bone pain when movement and changing position**
**Osteosarcoma**	**10–25, and >40**	**A rapidly expanding, painful chest wall mass (usually in ribs and sternum)**
**Ewing sarcoma (Askin’s tumor)**	**14 (most in children and sometimes in young adults)**	**Chest wall pain (usually a solitary tumor in ribs, sternum, scapula, clavicle, or paravertebral region)**
**Chondrosarcoma**	**>50**	**An enlarging, painful, anterior chest wall mass (common involvement of costochondral arches or sternum)**
**Breast carcinoma in men**	**65–67**	**A painless firm mass**
**Metastatic neoplasm**	**Usually in adults**	**Chest wall pain (might include pathologic fracture)**

N/A: Not Applicable.

In this case, the myofibroma does not result in changing of the external appearance or painful motion. On MRI examination, a unique postgadolinium feature of myofibromas is a central area of hypointensity surrounded by peripheral hyperintense enhancement [4]. However, in our case, the mass is heterogeneously hyperintense on T2 pregadolinium and inhomogeneous on contrast enhancement with gadolinium injection. A malignant neoplasm cannot be totally excluded. Clinically, diagnosis options include an excisional biopsy, a simple excision, or needle aspiration biopsy. However, needle aspiration biopsy was difficult to perform in our case because the mass lesion was deeply located and hindered by the right serratus anterior muscle, latissimus dorsi muscles and scapula. According to the reports, the myofibromas generally responded well to surgical treatment alone [1,2].

## Conclusions

In summary, although myofibroma and myofibromatosis typically present in children and babies, our case introduces myofibroma as a lesion located in the chest wall and presented with chest pain of an adult man. To the best of our knowledge, this is the first report of a myofibroma in an adult man that involved the chest wall. Although myofibroma rarely occurs in the chest wall and adults, the possibility of a chest wall tumor presenting with chest pain must be considered.

## Consent

Written informed consent was obtained from the patient for publication of this case report and any accompanying images. A copy of the written consent is available for review by the Editor-in-Chief of this journal.

## Abbreviations

CT: Computed tomography; MRI: Magnetic resonance imaging.

## Competing interests

The authors declare that they have no competing interests.

## Authors’ contributions

Both authors participated in the patient’s diagnosis and medical management. C-LC drafted the first version of the manuscript and HC drafted the revised manuscript. Both authors have read and approved the manuscript.
